# Stakeholder-centric explanations for black-box decisions: an XAI process model and its application to automotive goodwill assessments

**DOI:** 10.3389/frai.2024.1471208

**Published:** 2024-10-24

**Authors:** Stefan Haas, Konstantin Hegestweiler, Michael Rapp, Maximilian Muschalik, Eyke Hüllermeier

**Affiliations:** ^1^Institute of Informatics, LMU Munich, Munich, Germany; ^2^BMW Group, Munich, Germany; ^3^Munich Center for Machine Learning, Munich, Germany

**Keywords:** eXplainable AI (XAI), prescriptive machine learning, decision support systems (DSS), SHapley Additive exPlanations (SHAP), goodwill assessment

## Abstract

Machine learning has made tremendous progress in predictive performance in recent years. Despite these advances, employing machine learning models in high-stake domains remains challenging due to the opaqueness of many high-performance models. If their behavior cannot be analyzed, this likely decreases the trust in such models and hinders the acceptance of human decision-makers. Motivated by these challenges, we propose a process model for developing and evaluating explainable decision support systems that are tailored to the needs of different stakeholders. To demonstrate its usefulness, we apply the process model to a real-world application in an enterprise context. The goal is to increase the acceptance of an existing black-box model developed at a car manufacturer for supporting manual goodwill assessments. Following the proposed process, we conduct two quantitative surveys targeted at the application's stakeholders. Our study reveals that textual explanations based on local feature importance best fit the needs of the stakeholders in the considered use case. Specifically, our results show that all stakeholders, including business specialists, goodwill assessors, and technical IT experts, agree that such explanations significantly increase their trust in the decision support system. Furthermore, our technical evaluation confirms the faithfulness and stability of the selected explanation method. These practical findings demonstrate the potential of our process model to facilitate the successful deployment of machine learning models in enterprise settings. The results emphasize the importance of developing explanations that are tailored to the specific needs and expectations of diverse stakeholders.

## 1 Introduction

With the growing access to large amounts of data and the widespread availability of computational resources, the idea of using *machine learning* (ML) methods to guide human experts toward more rational, objective, and accurate decisions, rather than relying solely on their experience and intuition, becomes increasingly prevalent in many application domains. However, in high-stake domains, where decisions can come with severe consequences, there is often a reluctance to use ML methods. For example, this includes applications in healthcare, where decisions may significantly impact human lives, and use cases in finance or industry that come with the risk of economic loss (Burkart and Huber, 2021; Adadi and Berrada, 2018). Concerns about adopting ML-driven technology are often attributed to the black-box characteristics of high-performance models, such as ensembles of decision trees or neural networks, which cannot easily be inspected, verified, and rectified by humans. Motivated by safety-critical applications, where the ability to understand a model's behavior is crucial for its successful adoption and acceptance by humans, there is a growing demand for *explainable artificial intelligence* (XAI). Besides the development of novel and inherently interpretable supervised ML methods (e.g., Rudin, 2019; Lou et al., 2012, 2013; Ustun and Rudin, 2016), this direction of research has led to various algorithmic solutions aimed at increasing the transparency of existing black-box approaches through *post-hoc* explanations (e.g., Ribeiro et al., 2016, 2018; Lundberg and Lee, 2017; Guidotti et al., 2018a; Plumb et al., 2018; Ming et al., 2018), which explain the inner workings and decision-making process of a trained machine learning model, after the model has already been developed and deployed. One can further distinguish between model-specific or model-agnostic methods, where an explanation method is limited to a specific model class or is model independent, respectively (Burkart and Huber, 2021). In the following, we focus on the latter, as model-agnostic, *post-hoc* approaches allow us to improve on existing models, which are proven to provide robust and accurate predictions.

Our research is driven by a real-world application in the automotive domain, where an ML-based system should support the assessment of goodwill requests. The goodwill process enables car dealers to request monetary compensation for reparations from the manufacturer on behalf of their customers. It qualifies as a high-risk business use case, as bad decisions either negatively affect customer satisfaction or harm the manufacturer's financial interests. Since the manual assessment of goodwill requests is tedious and time-consuming as automotive manufacturers receive up to several tens of thousands of goodwill requests per year, ML provides a tempting opportunity to reduce manual efforts and save costs. Moreover, due to the availability of tens of thousands or even hundreds of thousands of past goodwill requests and their respective outcome, supervised machine learning techniques can be used and have been shown to succeed in closely capturing expert decisions (Haas and Hüllermeier, 2023). However, despite these promising results, the opaqueness of existing models , due to their complex non-interpretable hierarchical structure and usage of gradient boosting, prevents their employment in practice. It is considered a significant limitation by stakeholders, who naturally want to limit the risk of unexpected behavior and therefore demand auditability of the models.

The explanatory needs of different stakeholders are typically context-dependent and may vary between different interest groups. For this reason, a single explanation method cannot always be expected to satisfy the requirements of different stakeholders across a wide variety of applications. As a result, the task of developing interpretable supervised ML systems can only partially be solved from an algorithmic perspective. Instead, it must be considered with high priority during a system's design, development, and evaluation phases. To our knowledge, no complete framework for developing XAI solutions deliberately tailored to different interest groups has yet been proposed in the literature. Instead, as elaborated in Section 2 below, existing publications tend to focus on specific aspects of the topic, such as algorithms, technical evaluation methods, visualization approaches, or user studies. As an important step toward closing the gap between these different research directions, we investigate an end-to-end approach considering all necessary steps for developing an XAI system, starting with stakeholder identification and requirements engineering over implementation to evaluation and user feedback. In summary, the contributions of our work are the following:

- In Section 3, we first discuss the real-world problem of automated goodwill assessment that further motivates the need for explainable ML systems in high-stake domains.
- In Section 4, we propose a streamlined and holistic process model for developing *post-hoc* explainable decision support systems based on findings from interdisciplinary literature and practical considerations.
- In Section 5, we demonstrate how the proposed process model can be applied to the previously introduced real-world scenario and validate its usefulness to meet the explanatory needs of different stakeholders.

By following a stakeholder-centric approach to XAI, we aim to overcome the reluctance to use ML-based solutions in an exemplary business context and hypothesize that our results can be transferred to similar domains. Concretely, we want to validate whether following this process model helps us to overcome the skepticism of ML usage in our exemplary high-stake business process. In detail, we would like to know whether increased stakeholder-centered transparency through XAI methods actually eases the introduction of ML into this high-stake process.

## 2 Related work

As our goal is to propose a process model deeply rooted in the XAI literature, this section provides a broad overview of existing work on the topic. Developing and evaluating transparent ML systems is an interdisciplinary effort, ranging from machine learning over human-computer interaction and visual analytics to the social sciences. Consequently, several comprehensive surveys exist that aim to consolidate this vast field of research (e.g., Burkart and Huber, 2021; Dwivedi et al., 2023; Minh et al., 2022; Adadi and Berrada, 2018; Guidotti et al., 2018b; Ali et al., 2023; Longo et al., 2024). However, these surveys are far from being an actionable guidance for practitioners in terms of how to approach the topic of XAI in concrete (high-stake) domain implementations.

Nevertheless, many existing publications focus on specific aspects of XAI instead. On the one hand, this includes work on technical aspects of the topic, such as the algorithmic details of different evaluation methods (e.g., Mc Grath et al., 2018; Molnar et al., 2020) and approaches for evaluating them quantitatively (e.g., Lopes et al., 2022; Bodria et al., 2021; Doshi-Velez and Kim, 2017). On the other hand, because XAI's primary goal is to satisfy the explanatory needs of human users and overcome their skepticism about ML-based technology, research efforts have also been devoted to relevant aspects of human-computer interaction. Among others, contributions in this particular direction include studies on how knowledge about ML models should be presented to users visually (e.g., Hudon et al., 2021). In addition, the challenges of gathering feedback from users and measuring their satisfaction in ML systems are also frequently addressed in user studies (e.g., Kenny et al., 2021). A survey-based methodology for guiding the human evaluation of explanations with the goal to simplify human assessments of explanations is presented by Confalonieri and Alonso-Moral (2024). However, again, this study only focuses on the human evaluation part, neglecting all other parts of XAI system development.

The focus on stakeholder perspective and needs is, amongst others, emphasized by Langer et al. (2021). To our knowledge, Vermeire et al. (2021) are the only ones that address the problem of bridging the gap between stakeholder needs and explanation methods from a practicable and actionable angle. Concretely, they propose explanation ID cards and questionnaires to map explanaibilty methods to user needs. However, their methodology does not cover further technical or user-centered assessments of the matched explanation methods, which may be required in high-stakes settings to ensure reliable and useful explanations. Furthermore, an empirical validation of their proposed method is still missing. In line with our work, XAI tools and processes found in the literature are mapped to common steps in software engineering in Clement et al. (2023). Though the different software engineering phases also appear reasonable in an XAI context, starting out from requirements analysis over design implementation and evaluation over to deployment, the phases rather serve as a structure for the survey than an actionable methodology for practitioners developing XAI systems. Similarly, in Amershi et al. (2019), a general software engineering approach for developing ML systems is derived from practical experience. However, it does not cover any aspects of transparency. A unified framework for designing and evaluating XAI systems, based on a categorization of design goals and corresponding evaluation measures according to different target groups, is presented in Mohseni et al. (2021). However, the framework lacks guidance in terms of concrete XAI method selection. Moreover, it is worth mentioning that the European Commission provides a loose set of requirements for trustworthy AI systems (Floridi, 2019). In addition to the valuable insights provided by the publications mentioned above, we also rely on the taxonomies outlined in Burkart and Huber (2021), Adadi and Berrada (2018), Guidotti et al. (2018b), Arrieta et al. (2020), Meske et al. (2022), and Markus et al. (2021).

Similar to our work, research on XAI is often motivated by specific applications and use cases. Case studies have been conducted in many domains, including the insurance industry (van Zetten et al., 2022), finance (Purificato et al., 2023; Zhu et al., 2023), the public sector (Maltbie et al., 2021), auditing (Zhang et al., 2022), and healthcare (Gerlings et al., 2022). Usually, these studies can be grouped into either purely technically focused studies, without end-user or domain expert involvement, (e.g., Zhu et al., 2023; Orji and Ukwandu, 2024) or studies where feedback regarding the explanations and their comprehensibility is also collected from domain experts or end-users (e.g., van Zetten et al., 2022; Maltbie et al., 2021). The study presented by Baum et al. (2023) stands out as it follows the conceptual model presented by Langer et al. (2021), which considers explanation approaches and information as a means to satisfy different stakeholder desiderata (e.g., interests, expectations, needs, etc.) in particular contexts. Baum et al. adapt this conceptual model in a more practical way by starting with the different stakeholders, which they consider the main context of the explanation, and their particular needs. Based on this, explanation information and concrete XAI methods can then be derived. However, the study lacks empirical validation.

Moreover, beyond these logical paradigms, there are cognitive semantic interpretations that address non-formalisable (black box) aspects of AI. XAI can be conceptualized as a hybrid space where human and machine cognition interact distinctly. For instance, Miller (2019) discusses the importance of cognitive approaches in XAI, highlighting how cognitive semantics can make AI systems more understandable and trustworthy. Several researchers have proposed unique convergent methodologies from a wide array of disciplines (e.g., cognitive modeling, neural-symbolic integration) to ensure XAI's purposefulness and sustainability.

Due to most of the presented works only focusing on specific aspects of XAI and the lack of a coherent methodological framework for XAI system development, which was, for instance, amongst others acknowledged by Bhatt et al. (2020), Langer et al. (2021), and Vermeire et al. (2021), we see an urgent need for a holistic XAI system development process model providing guidance to deploy XAI systems in practice. Even more, as Bhatt et al. (2020) notice that the majority of XAI deployments are not for end users affected by the model but rather for machine learning engineers, who use explainability to debug the model itself, which shows a severe gap between explainability in practice and the goal of transparency for all involved stakeholders.

## 3 Application domain

As mentioned earlier, the process model proposed in this work is motivated by a real-world application in the automotive domain, where an ML system should support human decision-makers. In the following, we outline the requirements of said application and motivate the need for explainable machine learning models in the respective domain.

### 3.1 Warranty and goodwill in the automotive industry

Warranty and goodwill are essential aspects of after-sales management in the automotive industry. Vehicles are often costly, so customers have high expectations regarding the reliability of these products. Even if significant efforts are put into quality control, due to the vast number of vehicles sold by *original equipment manufacturers* (OEMs), many warranty claims and goodwill requests must unavoidably be dealt with each year.

Warranty—in contrast to goodwill—is a legal obligation of the OEM. If a customer notices a defect within a legally defined period of time, the manufacturer must rectify the problem at his own expense. If no adequate solution can be provided, the customer may even have the option to withdraw from the purchase contract. However, it should be noted that the exact legal provisions for warranty may vary from country to country.

Goodwill describes an OEM's willingness to offer repairs, replacements, or financial compensations in the event of defects beyond the scope of warranty. There are no legal obligations here, i.e., an OEM can freely choose a strategy according to which goodwill requests should be handled. However, many manufacturing companies consider goodwill a vital tool to increase customer loyalty. From an OEM's point of view, compensations paid in response to goodwill requests can be understood as marketing investments that may positively affect the loyalty of its existing customers.

Since the duties that come with warranty are clear and legally binding, it is relatively straightforward to process warranty claims automatically, e.g., via rule-based systems. Only in difficult cases, or if the warranty process should be audited, it might be necessary for human experts to check individual claims manually. When we refer to manual expert activity within this study, we refer to *expert judgement*, in which single automotive after-sales experts or assessors leverage the accumulated knowledge, skills, and intuition they have developed over time to make a decision. This is in contrast to *networked expertise*, where the skills and knowledge of multiple experts are combined, or a more guided approach like *quality function deployment (QFD)*. Unfortunately, in the case of goodwill assessments, it is much more challenging to achieve a high degree of automation. For example, even though the car manufacturer employs a rule-based system to deal with goodwill requests in an automated manner, a large fraction of the received requests require a manual examination by human experts. Among 688, 879 goodwill requests considered in Haas and Hüllermeier (2023), only 349, 488 (50.73%) could be processed automatically, whereas 339, 391 (49.27%) demanded a manual assessment. Consequently, there is great potential to increase the degree of automation in the goodwill assessment process through machine learning techniques.

### 3.2 The use of machine learning in the goodwill assessment process

**Figure 1:**
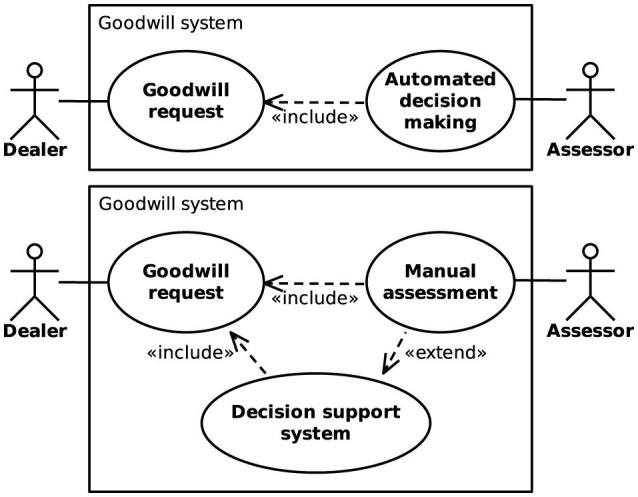
Integration of machine learning models into a goodwill assessment system using automated decision-making **(Top)** or a decision support system **(Bottom)**.

Supporting human assessors responsible for goodwill decisions through machine learning techniques is appealing from an OEM's perspective, as it can potentially reduce labor costs and foster a standardized goodwill strategy. Unlike decisions made by humans, which are often based on personal experience and intuition rather than being purely rational, assessments provided by ML models are deterministic. This helps to prevent cases where similar goodwill requests result in vastly different responses, which may damage the OEM's reputation. Figure 1 illustrates two different approaches considered in Haas and Hüllermeier (2023) for integrating ML models into the goodwill assessment process. The models can either be used for *automated decision-making* (ADM), where goodwill requests are processed automatically without human intervention, or as a *decision support system* (DSS), which merely provides recommendations to human experts and keeps them in control of the final decision. Whereas, ADM has a greater potential for cost savings, it does also come with a higher risk of incorrect decisions than the DSS approach, since no human supervision takes place.

Regardless of whether an ADM or a DSS approach is pursued, we consider the problem of providing automated goodwill decisions as a *prescriptive machine learning* problem, a term that has recently been coined by Hüllermeier (2021). It emphasizes differences between the tasks of predicting an outcome and prescribing some sort of action or decision in a certain situation. The former is commonly considered in the standard setting of supervised learning, which assumes a kind of objective ground truth (used as a reference to assess the prediction). In the prescriptive setting, on the other side, there is normally nothing like a “true” or “correct” decision or action—in general, not even the optimality of a prescription can be verified retrospectively, because consequences can only be observed for the one decision made, but not for those other actions that have not been taken.

This lack of ground truth is inherent to goodwill decisions, too, as it cannot be guaranteed that decisions made by experts in the past have always beneficial regarding the OEM's business strategy in the long run. Nevertheless, mimicking the behavior of experts appears to be a natural strategy, as historical data $D=\left\{\left({\vec{\text{x}}}_{1},y_{1}\right),\ldots ,\left({\vec{\text{x}}}_{n},y_{n}\right)\right\}$, which incorporates information about goodwill requests ∈X and corresponding decisions y∈Y, can easily be used for supervised machine learning. On the one hand, a goodwill request is represented in terms of several *features*. They describe the properties of a vehicle, such as its age, mileage, or whether it was serviced regularly. In addition, they may provide information about a defect that was encountered, including the type of malfunction and the expected repair costs. On the other hand, the possible outcomes of a goodwill assessment depend on the OEM's business strategy. For example, BMW requires assessors to decide for a percentage between 0%, in which case the manufacturer does not offer any compensation, and 100%, which means that the manufacturer fully bears the repair costs. To support the work of the assessors at BMW, Haas and Hüllermeier (2023) propose an ordinal classification method that models the outcome of goodwill decisions in terms of the compensation (multiples of 10%) as target variable *y* ∈ {0%, 10%, …, 100%}.

### 3.3 The need for explaining automated goodwill decisions

The previously mentioned ordinal classification method, developed at BMW and discussed in detail in Haas and Hüllermeier (2023), can be considered a *black-box model*. Even though it is able to achieve high accuracy compared to the historical decisions of human assessors, the model's opaqueness poses several challenges for its successful adoption in a business context. Due to its complexity originating from the usage of gradient boosted trees in combination with a hierarchical cost-sensitive framework (Haas and Hüllermeier, 2023), the model can neither be analyzed by human experts as a whole, nor does it provide any information about why certain decisions have been made. This leads to several issues regarding the acceptance and trustworthiness of the automated goodwill system. First, the lack of transparency impedes the ability of domain experts to audit the model and ensure that it adheres to the OEM's goodwill strategy. Second, because no reasons are given for a particular decision, it is hard to reason about cases where the system and human assessors disagree. This makes it difficult to provide valuable feedback that may help to improve the model and hinders the discovery of inconsistencies or biases in human decision-making.

**Figure 2:**
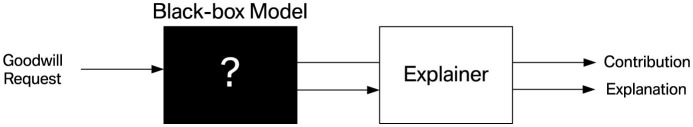
Model-agnostic *post-hoc* explainer for the black-box goodwill decision model.

Nevertheless, modern black-box models are valued for achieving state-of-the-art performance. Moreover, there is no legal obligation in goodwill for complete transparency of the assessment process. In settings like these, a solution that overcomes the aforementioned shortcomings while retaining the existing model is desirable. This motivates the use of *post-hoc explanation methods* that can provide insights into an existing black-box model. In particular, model-agnostic explanation approaches are appealing in this regard. They are intended to work with any ML model, regardless of the technical principles it relies on. Figure 2 provides a high-level overview of the interaction between a black-box model and an associated *post-hoc* explainer that aims to clarify the model's behavior. Section 4.2 discusses the characteristics and goals of commonly used explanation methods in more detail.

**Figure 3:**
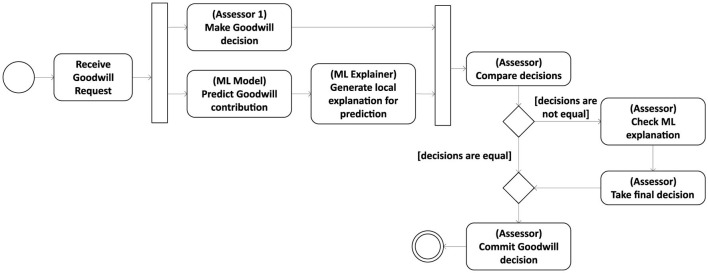
Human-in-command approach for an explainable decision support system.

Due to the unavoidable risk of incorrect decisions in an ML-driven assessment process, in the following, we focus on using machine learning models in the context of decision support systems rather than for automated decision-making. Integrating explanation methods into a DSS, which by design requires human practitioners to closely interact with the automation system, facilitates its employment in high-stake domains and opens the door to the *human-in-command* (HIC) approach (Floridi, 2019) outlined in Figure 3. In this approach, a goodwill assessor can consult an explainable decision support system to safeguard his or her decisions. The assessor and the ML model decide independently on a given goodwill request. If the recommendation provided by the latter differs from the manual assessment, the assessor must be able to obtain a human-understandable explanation for the model's outcome to decide whether it is appropriate to revise the own decision.

## 4 A process model for developing *post-hoc* explanation systems

**Figure 4:**
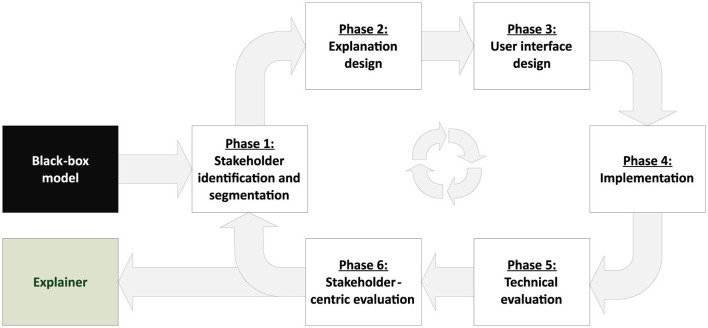
Iterative process model for developing an explainable decision support system, based on an existing black-box model.

As argued in Section 1, there is an urgent need for increased transparency and trust in black-box machine learning models to be used in high-stake domains. Among others, transparency and trust are two of the main goals of XAI (see, e.g., Burkart and Huber, 2021; Arrieta et al., 2020; Lipton, 2018; Fiok et al., 2022). However, selecting the best-suited XAI tools for a specific use case from the vast amount of available methods can be challenging. Usually, not all available solutions can satisfy the explanatory needs of stakeholders equally. Hence, a deliberate selection of suitable tools and a careful evaluation of feedback received from stakeholders is crucial to meet the expectations in an XAI system. For this reason, we propose a process model for developing an explainable decision support system (eDSS) using a *design-science-research* approach (Simon, 1988). An overview of the iterative procedure, including the individual phases it consists of, is shown in Figure 4.

The focus of the process model is to identify and validate suitable *post-hoc* XAI methods, which allow for turning an ML-based DSS into an eDSS. The process starts with an existing black-box model and the intended result is a *post-hoc* explanation system that is tailored to the problem domain and the explanatory needs of the system's stakeholders. The different phases of the proposed process model are, on the one hand, motivated by the XAI literature review presented in Section 2 and the herein identified gaps and requirements, but are also grounded in several complementary theoretical perspectives from the fields of stakeholder theory, human-computer interaction (HCI), and decision support systems (DSS), which further justify the phases themselves and their sequence. At the core of the process model is a strong emphasis on stakeholder engagement, which is informed by stakeholder theory (Freeman and McVea, 2005; Mitchell et al., 1997; Mahajan et al., 2023). Stakeholder theory posits that organizations should consider the needs and interests of all parties affected by their decisions and actions, not just their shareholders, which in turn leads to a broader perspective, long term sustainability, ethical considerations, shared value creation, and eventually a competitive advantage. In the context of XAI system development, this translates to actively involving diverse stakeholder groups, such as end-users, domain experts, policymakers, and management, throughout the design and evaluation process, which is also common sense in XAI research (Kim et al., 2024; Langer et al., 2021; Longo et al., 2024; Baum et al., 2023). For instance, Baum et al. (2023) consider the different stakeholders and their needs as the main context of XAI system development that needs to be elucidated first. The explanation design phase of the process model is informed by principles and theories from the field of human-computer interaction (HCI). Specifically, the model draws on research on cognitive fit (Vessey, 1991) and mental models (Johnson-Laird, 1983) to ensure that the explanations generated by the eDSS are aligned with the mental representations and information processing capabilities of the target end-users and stakeholders, and hence useful and actionable. Additionally, the stakeholder-centric evaluation phase is grounded in user-centered design approaches (Norman, 2002; Mao et al., 2005), which emphasize the importance of feedback from end-users to inform the design and refinement of interactive systems. By incorporating qualitative and quantitative assessments of stakeholder satisfaction and comprehension, the process model aims to develop explanations that are not only technically sound but also meaningful and useful to the intended users. There is also consensus in XAI research that a solid validation of an XAI system requires both a user-centered and a technical evaluation (Mohseni et al., 2021; Longo et al., 2024; Lopes et al., 2022). The overall structure of the process model, with its focus on developing an explainable decision support system, is informed by classic theories and frameworks from the field of decision support systems (Keen, 1980; Sprague, 1980). DSS research has long emphasized the importance of user involvement, information presentation, and the integration of human judgment with analytical models to support complex decision-making (Shim et al., 2002; Power, 2002). By adapting these DSS principles to the context of XAI, the proposed process model ensures that the resulting eDSS not only provides accurate predictions but also supports stakeholders in understanding, trusting, and appropriately using the ML-based decision support system (Turban et al., 2010; Arnott and Pervan, 2005). This is also in line with Burkart and Huber (2021), who suggest to consider three aspects for building a useful explanation system: *Who* should be addressed by the explanations, *what* aspects of an ML system should be explained, and *how* should the explanation be presented. In the following subsections, we elaborate on the individual phases of our process model related to these fundamental questions.

### 4.1 Phase 1: Stakeholder identification and segmentation

Complex computer systems typically have several stakeholders that finance, design, build, use, or audit the system. Developing an eDSS should therefore start with identifying these interest groups, which may have varying expectations in the system and demand for different types of explanations (Gerlings et al., 2022; Kim et al., 2024). In the literature, the stakeholders of ML-based systems are usually separated into three main groups (see, e.g., Burkart and Huber, 2021; Mohseni et al., 2021; Arrieta et al., 2020; Meske et al., 2022), albeit named inconsistently. We rely on the terminology introduced by Hong et al. (2020):

- *Model consumers* or users are the persons affected by the decisions of an ML system. They can interact with the system passively or actively. In the former case, decisions are merely presented to the users, e.g., informing them about the approval or rejection of a loan. In the latter case, the predictions and explanations provided by the system should support human decision-makers, e.g., the person in charge of approving or rejecting a loan. In general, model consumers are not necessarily technical experts. And if they interact with a system passively, they can most likely not be considered domain experts.
- *Model builders* are responsible for developing and operating an ML model. They are proficient in ML but typically not domain experts.
- *Model breakers* are domain experts who have the necessary knowledge to verify that a model behaves correctly and meets the desired goals from a business perspective. However, they are usually not ML experts.

### 4.2 Phase 2: Explanation design

**Figure 5:**
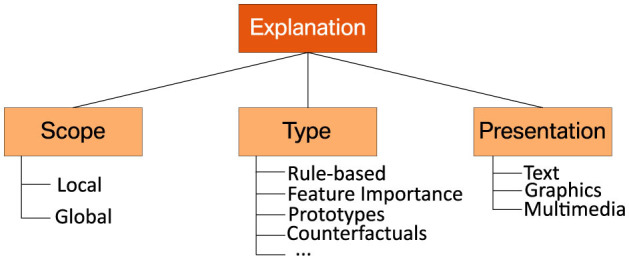
Taxonomy of commonly used explanation methods and presentation forms.

Once the interest groups of a system have been identified, the next step is to determine which aspects of an ML system need to be explained to each. Following Clement et al. (2023), we refer to this process as the “explanation design phase”. Possible explanations can hereby differ in their scope and the technical principles they are based on Burkart and Huber (2021). As the usefulness of available explanation methods depends on the application context and the needs of the stakeholders, their individual goals and limitations must be considered for a well-informed choice. Figure 5 provides an overview of the technical differences between commonly used explanation methods discussed below. In the literature, different XAI methods are often characterized by the scope of the explanations they provide (see, e.g. Burkart and Huber, 2021; Adadi and Berrada, 2018; Molnar et al., 2020; Bodria et al., 2021):

- *Global explanations* aim to provide a comprehensible representation of an entire ML model. Their goal is to make the overall behavior of a model transparent by capturing general patterns used by it.
- *Local explanations* focus on individual predictions provided by an ML system. They aim to disclose the reasons for why a particular decision has been made.

As previously mentioned, the preferred scope of explanations depends on the target audience and the application context. For example, product managers might be more interested in global explanations, as they allow them to verify a model's behavior by comparing the patterns it uses to their mental model. In contrast, human decision-makers might prefer local explanations, which can help them make specific decisions.

The most suitable explanation method also depends on the type of data used for training a model, such as tabular data, images or text (Bodria et al., 2021). As the application presented in Section 3 requires the handling of tabular data, we restrict ourselves to this particular scenario, where the following types of explanations are commonly used:

- *Rule-based* models and the conceptually related decision trees are often considered as inherently interpretable (Burkart and Huber, 2021). Hence, it is a natural choice to use rule-based representations for explaining black-box models (Guidotti et al., 2018a).
- *Feature importance* methods provide a ranking of the features found in the data, based on their contribution to a model's decisions (Ribeiro et al., 2016; Lundberg and Lee, 2017).
- *Prototypes* are the minimum subset of data samples that can be viewed as a condensed representation of a larger data distribution. Prototypes can either be obtained for general concepts found in the data or chosen based on their similarity to a particular example at hand (Bien and Tibshirani, 2011).
- *Counterfactuals* provide additional information about a model's predictions in the form of “what-if” scenarios. For example, they can expose the minimal changes of the input required to obtain a different outcome (Mc Grath et al., 2018; Molnar et al., 2020; Wachter et al., 2017). Unlike the other types of explanations listed above, counterfactuals cannot explain a model globally.

### 4.3 Phase 3: User interface design

Once the most suitable technical methods for explaining an ML model's behavior to stakeholders have been identified, an appropriate representation of the explanations must be found. Following Clement et al. (2023), we refer to this phase as the “user interface design”. As the form in which explanations are presented to the target audience may significantly influence their intelligibility and usefulness, it is crucial to our process model. Burkart and Huber (2021) distinguish between the following types of representations:

- *Textual explanations* rely on natural language to inform the user, e.g., using complete sentences or bullet lists to justify why a particular decision was made. Textual descriptions can be intuitive because humans tend to explain their decisions verbally.
- *Graphical explanations* make use of visual illustrations, such as plots or diagrams. They may convey complex information in a condensed manner and are supported by many software libraries (e.g., Nori et al., 2019).
- *Multimedia explanations* may combine several types of representation forms, including text, graphics, audio, and video.

Again, the type of representation that best fits the stakeholders' explanatory needs is context-dependent. For example, human decision-makers who must present decisions to customers might prefer a textual description over a visual one. If the information provided by an XAI system is given in text form, they can more easily adopt the explanation and verbally communicate it to the customer. This might ease their work significantly compared to a graphical representation, where they must first extract the essential information and reformulate it in an appropriate verbal response.

### 4.4 Phase 4: Implementation

After one has decided on XAI methods and corresponding representation forms that are most promising to fulfill the demands in a particular use case, the technical groundwork must be laid for further testing the pursued solution. Generally, this requires implementing the selected explanation methods, integrating them with an existing ML model, and deploying the resulting software. As these steps highly depend on the infrastructure used in a particular application context, it is impossible to provide general advice on the implementation phase of our process model. So, instead, we continue with the technical and user-centric evaluation to be conducted afterward.

### 4.5 Phase 5: Technical evaluation

**Figure 6:**
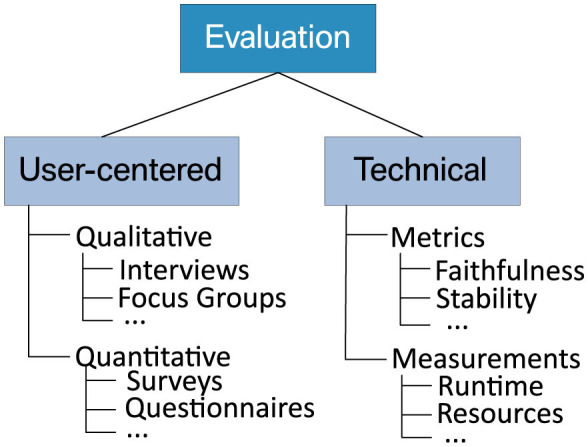
Taxonomy of technical and stakeholder-centric evaluation methods.

In the literature, there is a consensus that the evaluation of an XAI system should comprise a technical and a stakeholder-centric evaluation (Lopes et al., 2022; Mohseni et al., 2021). This obligation is also underpinned by several case studies that employ qualitative and quantitative methods to assess the correctness and suitability of explanations in a given setting (e.g., van Zetten et al., 2022; Maltbie et al., 2021). Moreover, Doshi-Velez and Kim (2017) provide a taxonomy for categorizing XAI evaluation methods. They distinguish between “functionally grounded” approaches based on formally defined metrics and “application-” or “human-grounded” techniques, where humans rate the quality of explanations. Similarly, Figure 6 provides an overview of commonly used evaluation techniques that we consider technical or stakeholder-centric. In the following, we first focus on the former before we continue with the latter in the subsequent section.

Technical evaluation methods aim to ensure the soundness of explanations. This is crucial because faulty behavior of an XAI system may fool an expert into making wrong decisions with severe consequences in high-stake domains. Bodria et al. (2021) highlight the following metrics for safeguarding the functional correctness of explanations:

- *Stability* validates how consistent the explanations provided by an XAI method are for similar examples.
- *Faithfulness* assesses how closely an explanation method can approximate the decisions of a black-box model.

Additional evaluation metrics for use in XAI are constantly proposed (see, e.g., Belaid et al., 2022 for a more extensive overview). For example, we also take runtime and usage of computational resources into account in Section 5.5.

### 4.6 Phase 6: Stakeholder-centric evaluation

A conceptionally sound and, according to technical criteria, properly working *post-hoc* explanation system might still not entirely fulfill the expectations and demands of individual stakeholders. For this reason, an essential building block of our process model is to evaluate an XAI system's usefulness with regard to the previously identified interest groups. As stressed by Lopes et al. (2022), this second evaluation phase aims to ensure the system's trustworthiness, measure the users' satisfaction, and verify the understandability and usability of the provided explanations. Because a purely technical approach cannot assess these qualitative goals, Doshi-Velez and Kim (2017) emphasize the need to gather feedback from humans working with the system in a real-world setting. When conducting such a user study, the technical background and (possibly lacking) domain knowledge of different interest groups must be considered to allow a realistic assessment of the explanations' comprehensibility. After all, if an explanation is not understandable from an end-user's perspective or is communicated inadequately, this may hamper the ML system's usefulness and trustworthiness.

One challenge of user-centric studies is to gather feedback from humans about their, most likely subjective, opinions regarding predefined goals in a structured and comparable way. Unfortunately, transcripts of personal interviews or reports written by participants (see, e.g., van Zetten et al., 2022; Maltbie et al., 2021; Cahour and Forzy, 2009) can be difficult to analyze. As an alternative, we advocate using Likert-scale questionnaires (see, e.g., van Zetten et al., 2022; Bussone et al., 2015), as discussed in Section 5.6.

## 5 Case study on automotive goodwill assessment

To demonstrate how the process model introduced in the previous section can be used in practice, we applied it to the application outlined in Section 3. Our goal was to extend an existing black-box model for goodwill assessment in the automotive domain with a *post-hoc* explanation system tailored to the needs of different stakeholders. Moreover, evaluating a conceptual method artifact and its effect on a real-world situation through a case study is a common evaluation method in design science research (Peffers et al., 2012).

### 5.1 Phase 1: Stakeholder identification and segmentation

According to the first step of our process model, we started by identifying the different stakeholders of the goodwill system. Based on our knowledge about the business use case at hand and discussions with representatives from potential interest groups in focus group meetings, we identified the following stakeholders:

- *IT specialists* employed by the OEM are responsible for developing, maintaining, and operating the goodwill system and its underlying ML model. They are technical experts but not domain experts.
- *Business specialists at the OEM* steer and control the company's global goodwill strategy from a business perspective and are responsible for all operational tasks. They are domain experts but not technical experts. Moreover, they collaborate closely with business specialists from *national sales companies* (NSCs), as described below.
- *Business specialists at NSCs* define guidelines for handling goodwill requests specific to a particular market and supervise the assessors operating in the respective area. They work closely with the parent organization's business specialists and, similar to the latter, are domain experts rather than technical experts.
- *Assessors* are domain experts who decide if the OEM should contribute to the costs of individual goodwill requests. Their decisions are based on the information available about a specific request and adhere to the guidelines established by business specialists. Moreover, assessors are active consumers of the ML system's recommendations.
- *Internal revisionists* audit the goodwill process. As goodwill does not come with legal obligations, they primarily ensure compliance with the OEM's strategic goals and guidelines.
- *Managers* responsible for quality control must ensure an efficient, fair, and transparent goodwill assessment process that benefits customer loyalty and, at the same time, keeps costs at an acceptable level.

**Figure 7:**
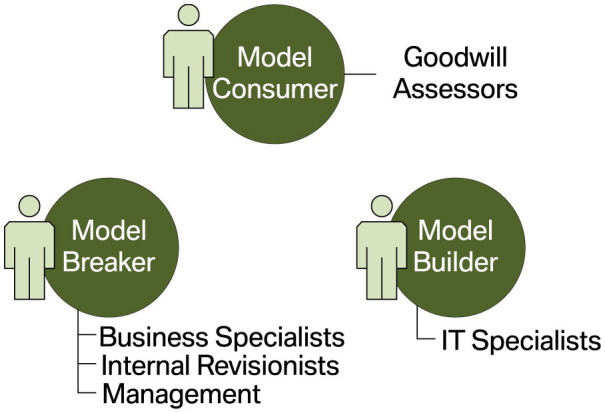
Segmentation of the goodwill system's stakeholders into interest groups.

Section 4.1 suggests assigning stakeholders to one of three groups: model consumers, model builders, and model breakers. The organizational structure outlined above matches this segmentation quite well. Assessors, who decide on goodwill requests and should actively be supported by the ML model, can be considered model consumers. IT specialists working on the ML system's technical aspects fulfill the roles of model builders. Finally, the responsibilities of business specialists at the OEM and NSCs are complementary. Like internal revisionists and managers, they are most interested in the ML system behaving consistently with their respective goals. Consequently, we consider them model breakers. Figure 7 illustrates the assignment of the goodwill system's stakeholders to distinct interest groups.

### 5.2 Phase 2: Explanation design

**Figure 8:**
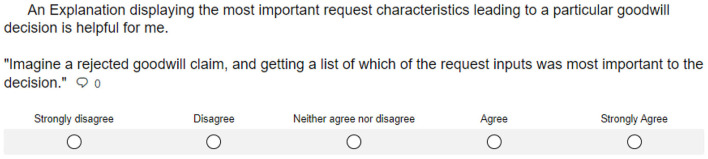
Question regarding the usefulness of local feature importance methods included in our explanation design survey.

After identifying and segmenting the goodwill system's stakeholders, our process model's next phase aims at identifying XAI methods that can satisfy their explanatory needs. When dealing with tabular data, we consider feature importance methods, prototypes, and rule-based explanations as technically suitable approaches. We conducted a five-point Likert-scale survey (Likert, 1932) to assess their usefulness regarding the stakeholders' expectations. In this survey, each explanation method was described on a non-technical level. In addition, we provided real-world examples of how the resulting explanations might be presented. Based on this information, we asked participants to what degree different explanations meet their requirements. For illustration, one of the questions included in the explanation design survey is shown in Figure 8.

**Figure 9:**
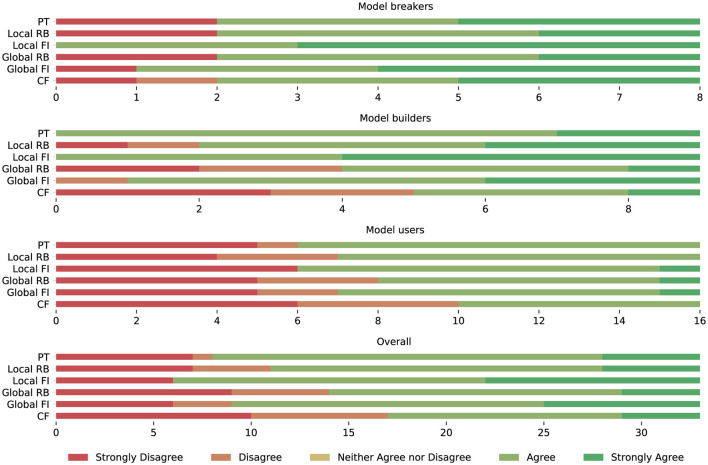
Frequency of agreement with the usefulness of prototypes (PT), rule-based explanations (RB), feature importance (FI), and counterfactuals (CF) by stakeholder group.

To ensure the understandability of the web-based survey by non-technical users and due to the limited availability of all stakeholders, it was iteratively refined together with model consumer and breaker team leads in focus group sessions before it was sent to the final pool of stakeholders. The survey was answered by 36 persons working on goodwill assessment in a single market where the decision support system was planned to be deployed. Among the participants were 16 model consumers, eight model breakers, and 12 model builders, representing the majority of the target audience in the considered market. Figure 9 shows how many participants from the different interest groups agreed with the usefulness of potential explanation methods according to a five-point Likert-scale.

We conducted a Shapiro-Wilk test (Shapiro and Wilk, 1965) to check for an approximately normal distribution of answers per group. For none of the stakeholder groups and explanation methods, the *p*-values exceeded the significance level α = 0.05. Consequently, the null hypothesis that the answers per group and method are normally distributed was rejected. Due to the non-normal data distribution, we conducted a non-parametric Kruskal-Wallis test (Kruskal and Wallis, 1952) to identify any statistically significant differences between the median answers of different stakeholder groups regarding the usefulness of individual XAI methods. The null hypothesis that the median is the same across all groups could not be rejected for counterfactuals and rule-based explanations (with α = 0.05). However, it was rejected for prototypes and feature importance methods. To discover which groups of stakeholders assess the usefulness of these explanation methods differently than the others, we finally conducted a *post-hoc* Dunn (1964) test. It revealed that the answers of the model users regarding the usefulness of local feature importance methods differ from those of the other groups to a statistically relevant degree (with α = 0.05). Table 1 summarizes the results of our analysis regarding the perceived helpfulness of explanation methods per stakeholder group. We conclude that all stakeholders of the goodwill system—especially model builders and breakers—consider local feature importance methods as the most promising XAI approach.

**Table 1:** Median agreement with the usefulness of XAI methods per stakeholder group.

**Explanation method**	**Stakeholder group**	**Useful?**
Local feature importance	Model breaker/builder	Strongly agree
	Model user	Agree
Global feature importance	All	Agree
Prototypes	All	Agree
Local rule-based	All	Agree
Global rule-based	All	Agree
Counterfactuals	All	Agree

### 5.3 Phase 3: User interface design

According to the previously conducted design study, all stakeholders of the goodwill system expect that explanations based on feature importance can best satisfy their requirements and provide valuable insights into the system's behavior. Hence, we focused on this particular type of explanation during the user design phase that lays the conceptual groundwork for the remaining steps of our process model. In particular, it requires identifying the information the selected approach can provide from a technical standpoint and exploring possibilities to present it to the user.

To explain goodwill decisions by disclosing the impact of individual features, we planned to employ *Shapley additive explanations* (SHAP) (Lundberg and Lee, 2017). This method derives feature importance scores from so-called *Shapley values* originating from game theory (Shapley, 1953). Unlike related methods such as LIME (Ribeiro et al., 2018) or permutation feature importance (Breiman, 2001), it provides theoretical properties well-suited for explaining ML models (Covert et al., 2020). As necessary in our use case, SHAP and the closely-related Kernel SHAP approximation method are model-agnostic *post-hoc* approaches that can be used with any black-box decision model. Moreover, an open-source implementation of these methods, including support for different visualizations, is available.^1^

SHAP provides local explanations in the form of an additive feature attribution function (Lundberg and Lee, 2017; Molnar, 2022)


$$g\left(z^{\prime }\right)=\phi_{0}+\overset{d}{\underset{j=1}{\sum }}\phi_{j}z_{j}^{\prime },$$

where *g* is the local linear surrogate explanation model and *z*′ ∈ {0, 1}^*M*^ is a data point represented by *M* binary features also called *simplified features*. In the simplified features, a value of 1 means that the feature is present whereas a value of 0 indicates absence. The importance of the *j*-th feature is specified by the absolut value of the Shapley value ϕ_*j*_ ∈ ℝ. Its sign indicates whether the feature has a positive or negative impact on the point prediction ŷ. This impact needs to be interpreted relative to a baseline ${𝔼}_{x}\left[\hat{f}\left(x\right)\right]$ that denotes the average of all model predictions.

In practice, the exact computation of Shapley values is often computationally infeasible, as 2^*d*^ feature subsets must be evaluated. To overcome this limitation, Kernel SHAP employs a sampling strategy for approximating Shapley values. For each data point *x* to be explained, the model is re-evaluated using a limited number of feature subsets (simplified features). Features that are missing from a subset (are set to 0) are withheld from the decision model. Unfortunately, individual feature values can only be removed from a data point if the model can handle missing values. Otherwise, they must be replaced by randomly sampled values to break the relationship between feature values and target variables (Covert et al., 2020). In case of tabular data, an absent feature equals replacement by a random feature value from the data. By adjusting the number of re-evaluations or samples, Kernel SHAP's computational demands and approximation quality can be traded off (see Section 5.5.3).

In the end, the linear explanation model *g* is trained by optimizing the following weighted sum of squared errors loss function *L*:


$$\begin{array}{l} L\left(\hat{f},g,\pi_{x}\right)=\underset{z^{\prime }\in Z}{\sum }{\left(\hat{f}\left(h_{x}\left(z^{\prime }\right)\right)-g\left(z^{\prime }\right)\right)}^{2}\pi_{x}\left(z^{\prime }\right) \end{array}$$

The estimated weights of the linear model *g* are then the Shapley values ϕ_*j*_ ∈ ℝ. $\hat{f}$ is the original model and *h*_*x*_ a helper function mapping simplified features to corresponding values from the actual instance *x* to be explained ($h_{x}:{\left\{0,1\right\}}^{M}\to {\mathbb{R}}^{M}$). π_*x*_ is the SHAP kernel providing a weight for each simplified feature vector. The basic idea is hereby to give small (few 1's) and large (many 1's) vectors the highest weights, as they provide the most information regarding the effect of individual features (isolated and total).

To obtain a global explanation for the model, the absolute Shapley values per *j*-th feature can simply be averaged over the data:


$$\begin{array}{l} I_{j}=\frac{1}{n}\overset{n}{\underset{i=1}{\sum }}|\phi_{j}^{\left(i\right)}| \end{array}$$

**Figure 10:**
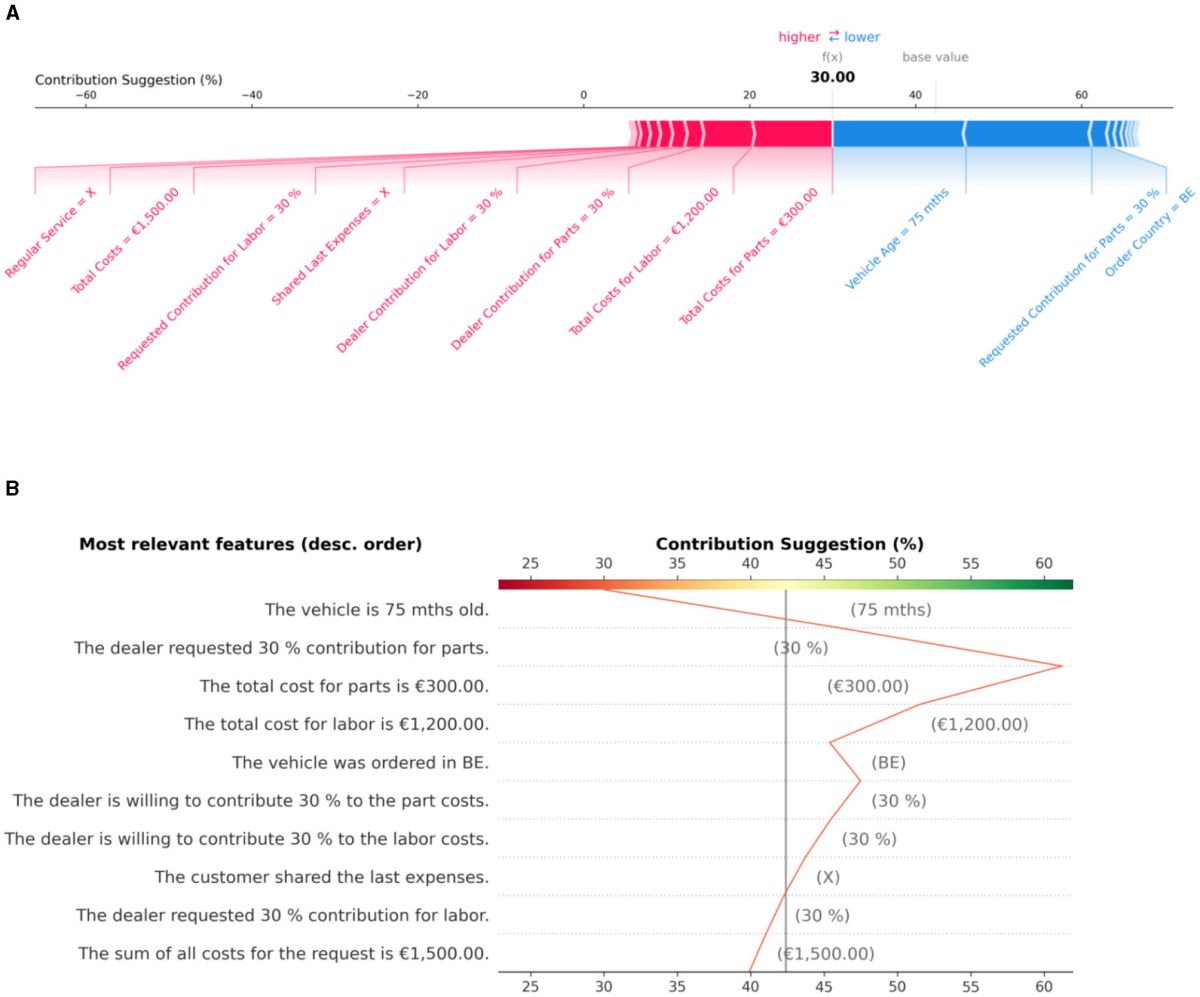
Graphical explanations of a goodwill decision based on feature importance. **(A)** The force plot displays feature values in favor of a higher contribution as red arrows, with their length indicating the magnitude of the contribution. Conversely, blue arrows represent feature values that contribute to a lower output value. Importantly, the importance of each feature value is always measured in relation to the base value in SHAP. **(B)** The text-enriched decision plot lists feature value descriptions in descending order of importance on the *y*-axis and the model's output on the *x*-axis. It is centered on the *x*-axis at the base value and shows through the oscillating line how the model's prediction changes through the contribution of the different feature values from bottom to top.

**Figure 11:**
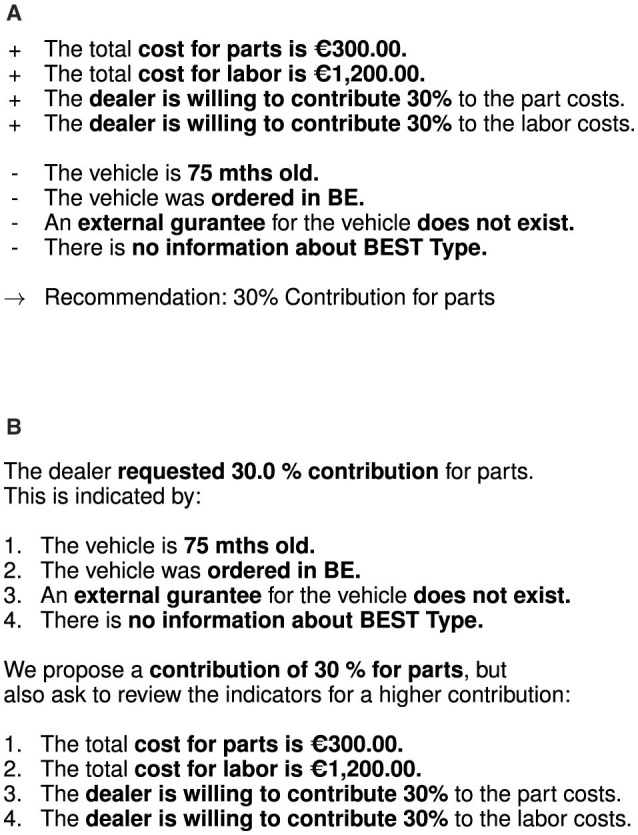
Textual explanations of a goodwill decision based on feature importance. **(A)** The text baseline approach displays the feature values contributing the most positively (+) as well as negatively (−) grouped and with descending importance, as well as the final recommendation by the model. **(B)** The decision-logic-enhanced text also groups the feature values with regards to their positive or negative contribution, but also puts the model's prediction into relation to what the dealer requested from the manufacturer on behalf of the end customer.

As outlined in Section 3, we utilize an ordinal classification method to decide on the percentage of goodwill costs to be taken by the OEM. In this context, features with negative Shapley values result in less compensation to be paid. In contrast, positive values correlate with a higher contribution. During the user interface design, we considered the following textual and graphical representations (see Figures 10, 11 for examples) to disclose the positive and negative factors that lead to a particular goodwill decision:

- We refer to a simple enumeration of the most influential features according to their Shapley values as the *text baseline*. It is restricted to features with positive (negative) values greater (smaller) than the quantile *q* = 0.85 (*q* = 0.15). The features are grouped by the sign of their Shapley values and sorted by their size in decreasing order.
- *Decision-logic-enhanced text* compares features supporting the financial claims that come with a goodwill request to those speaking against them or favoring a lower financial contribution. As before, only the most influential features favoring or contradicting a request are given in sorted order.
- *Force plots* visualize the contribution of individual features to a prediction based on their Shapley values. For this purpose, the positive or negative impact of each feature is shown relatively to the final prediction and the baseline value on a one-dimensional scale.
- Like the textual representations above, *text-enriched decision plots* provide a description of features sorted by their importance, albeit independently of whether they influence a prediction positively or negatively. However, similar to force plots, the contribution of each feature to the final prediction is shown graphically and put in relation to the baseline value.

### 5.4 Phase 4: Implementation

**Figure 12:**
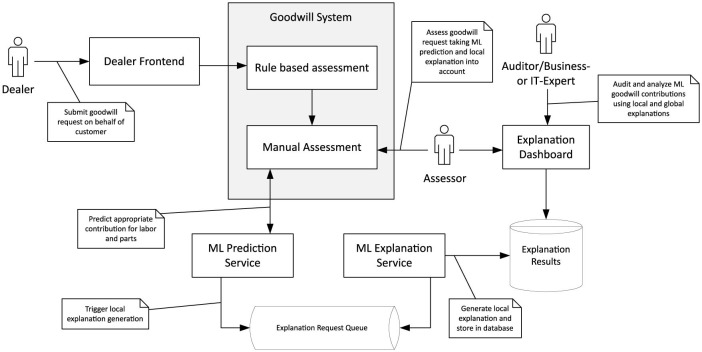
Software architecture of the decision support system for goodwill assessment.

Once the requirements in the explanation system have been identified, and one has settled for a technical approach that meets these demands, it must be implemented and integrated into the existing ecosystem. Figure 12 outlines the software architecture of the goodwill system. Dealers submit goodwill requests on behalf of their customers via the *dealer frontend*. As described in Section 3, requests are handled by a *rule-based assessment* if possible. Otherwise, a *manual assessment* must be performed. It starts with the invocation of the *ML prediction service* that recommends the compensation to be paid by the OEM for a particular goodwill request. In addition, the prediction service asynchronously triggers the *ML explanation service* by dispatching an explanation request to a FIFO queue monitored by the latter. Separating prediction and explanation into distinct micro-services is favorable as the execution of Kernel SHAP can be computationally costly and time-consuming. With micro-services, the underlying hardware can be scaled independently. Moreover, there is no need to provide explanations immediately after a new goodwill request arrives since it typically takes time until a human assessor can inspect them. Shapley values computed by the explanation service are stored in a central database. They are accessible through a web application called the *explanation dashboard*. Offering a standalone application for accessing explanations enables one to adjust to different stakeholder groups more flexibly. For example, assessors are most interested in explanations for pending goodwill requests. In contrast, other stakeholders like auditors or business experts might want to inspect goodwill decisions made in the past.

### 5.5 Phase 5: Technical evaluation

As the next step of our process model, a technical evaluation of the previously implemented explanation system should be conducted to ensure that it generates sound explanations. Such an evaluation is crucial as faulty explanations may trick human decision-makers into making wrong decisions. As part of our case study, we verify if the explanations based on Kernel SHAP fulfill two well-established evaluation metrics, namely *stability*, and *faithfulness* (Bodria et al., 2021; Belaid et al., 2022; Alvarez-Melis and Jaakkola, 2018; Rong et al., 2022). *Fidelity* (Bodria et al., 2021), another common evaluation metric, which measures how well an interpretable surrogate model reflects the predictions of the original black-box model, is given by the Shapley value's *efficiency* property $\;\sum_{j=1}^{M}\phi_{j}=h\left(\vec{\text{x}}\right)-\text{𝔼}\left[h\left(\vec{\text{x}}\right)\right]$ (Lundberg and Lee, 2017), which states that the feature contributions must add up to the difference of the prediction for $\vec{\text{x}}$ and the average or base value ($\text{𝔼}\left[h\left(\vec{\text{x}}\right)\right]$). Hence, there is no need to assess this experimentally. In addition, to ensure that the implementation adheres to operational constraints, we measure the *computation time* and *memory consumption* needed to generate explanations. The literature lists many more metrics like *completeness, actionability, compactness, interpretability*, and *plausibility*, among others (Markus et al., 2021; Zhou et al., 2021). However, quantifying them can be challenging without incorporating user feedback, as they often involve subjective judgments and context-specific considerations that are not easily captured through technical means alone. That's why we focus on the established technical key metrics *stability* and *faithfulness* for Kernel SHAP here.

#### 5.5.1 Stability

The stability of an explanation in the context of machine learning models is a crucial concept that refers to how sensitive the explanation is to small changes in the model's input. Explanation stability is an important consideration because it helps assess the reliability and robustness of the explanations provided by a machine learning model. If the explanations are highly sensitive to minor input perturbations, it can raise concerns about the trustworthiness and consistency of the model's decision-making process.

Stability can be assessed in terms of the *Lipschitz constant*


$$\begin{array}{l} L_{x}=\underset{x^{\prime }\in N_{x}}{\max}\frac{||e_{x}-e_{x^{\prime }}||}{||x-x^{\prime }||}. \end{array}$$

The test instance for which an explanation should be provided is denoted by *x*, whereas *e*_*x*_ is the corresponding explanation in the form of Shapley values. We normalize both of these vectors by the sum of their elements. Moreover, $N_{x}$ denotes a neighborhood consisting of instances *x*′ similar to *x* (Bodria et al., 2021; Alvarez-Melis and Jaakkola, 2018).

Based on domain knowledge, we explore the neighborhood *x*′ of a test instance *x* by applying random changes to some of its numerical features. This procedure is carried out for *mileage* (±100) with an interquartile range (IQR) of 72, 017.25, *vehicle age in month* (±1) with an IQR of 26.0, *labor costs* (±10) with an IQR of 415.0, *parts costs* (±10) with an IQR of 1, 150.0, and *open time costs* (±1) with an IQR of 34.96. For these relatively small changes we do not necessarily expect any changes in the model's predictions or the corresponding explanations.

Table 2 shows the results of our stability evaluation. Large values indicate great instability, meaning that for similar inputs quite different explanations are generated. In addition to the stability, its mean, and its standard deviation, we report the fraction of test instances for which predictions have changed compared to its neighbors. Finally, the table also includes the fraction of instances for which the top-2, -3, and -5 most important features according to Shapley values have changed due to the perturbations in some numerical features of neighboring instances. We observe that explanations of goodwill contributions to labor costs are far more unstable than those related to part costs according to the Lipschitz constant. For both of these explainers, the top-2 and top-3 most important features remain unaffected for the vast majority of test instances. However, for about 50% of the instances the top-5 ranks change, which indicates the limitations of Kernel SHAP's stability. Nevertheless, we consider this explanation method to be stable enough for our use case, because of the small number of changes in predictions and top-2 feature importance rankings.

**Table 2:** Stability of the Kernel SHAP explainer over a subset of 100 test samples.

**Explainer**	**Stability**	**Prediction changes**	**Top-2 FI changes**	**Top-3 FI changes**	**Top-5 FI changes**
Labor	1,026.4 ±1,507.4	0.01	0.04	0.12	0.47
Parts	544.0 ±939.6	0.00	0.00	0.18	0.53

#### 5.5.2 Faithfulness

The faithfulness of an explanation assesses how well the explanation approximates the true behavior of the underlying black-box machine learning model (Alvarez-Melis and Jaakkola, 2018). It measures how well the explanation captures the actual decision-making process of the model, rather than just providing a simplified or approximate representation. When dealing with explanations based on feature importance, their faithfulness can be evaluated by using so-called *deletion curves* (Petsiuk et al., 2018). According to this method, feature values are removed from test instances successively, depending on the importance of the corresponding features. The values of the most important features are removed first and after each deletion the model's prediction error is measured. The intuition behind this procedure is the following: If a particular feature is considered highly important by a feature importance method, its removal should lead to a drastic increase in prediction error. In contrast, the prediction error should only slightly deteriorate if one of the least important features is removed. When removing multiple features with decreasing importance, this should cause the prediction error to increase monotonically. Unfortunately, the used black-box models cannot handle tabular data from which individual features have been removed. To overcome this limitation, we sample from the marginal feature distribution to simulate the removal of features as suggested by Covert et al. (2021).

**Figure 13:**
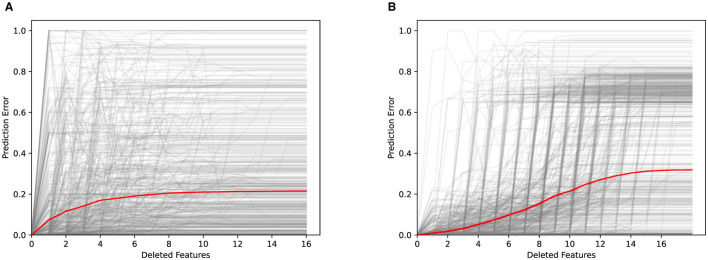
Deletion curves obtained for labor and part costs based on 578 randomly selected test instances. Features are removed by randomly sampling 10, 000 replacement values. The prediction error is computed as the relative error compared to the maximal possible error, which may vary depending on the prediction and therefore is normalized. **(A)** Labor. **(B)** Parts.

Figure 13 illustrates the faithfulness of the feature importance rankings that explain goodwill contributions to labor and part costs, respectively. In both cases, we observe that the removal of the most important feature already results in a significant change of the deletion curve. Moreover, the removal of additional features results in a monotonically increasing deletion curve until a plateau is finally reached. This testifies the faithfulness of the explanations provided by Kernel SHAP. For the labor costs, the average prediction error increases faster. However, in the limit, the prediction error is not affected as much as for the part costs.

#### 5.5.3 Runtime and memory consumption

The runtime and memory consumption of Kernel SHAP, apart from the underlying data and number of features, mainly depend on the size of the dataset and the number of times the model is re-evaluated, respectively, simplified features are sampled (nsamples parameter in the Kernel SHAP implementation) when explaining a prediction. In our use case, we have to deal with 26 features in total. As a result, the memory consumption of Kernel SHAP is the most limiting factor. We therefore enforced a memory limit at around 10 GB to keep the memory consumption at an acceptable level. As a result, the implementation was deployable on a high density cluster environment without the need to provide dedicated machines with larger main memory.

Table 3 shows the runtime and memory consumption of Kernel SHAP when generating explanations of the contribution to labor and part costs, respectively. The algorithm was provided with a dataset consisting of 100 instances. It was configured to perform 3, 000 re-evaluations or samples per explanation. In our use case, an average runtime of 25 s is acceptable, because explanations are provided to human assessors asynchronously instead of in real-time. The CPU utilization of ~1.7 millicores is moderate. The test was carried out on a machine with 8 vCPUs and 28 GB main memory.

**Table 3:** Maximum runtime and resource consumption of Kernel SHAP for 100 samples.

**Explainer**	**Max. runtime**	**Max. memory**	**Max. CPU**
Labor	24.92 s	9.99 GB	1,659 mc
Parts	24.15 s	9.02 GB	1,713 mc

### 5.6 Phase 6: Stakeholder-centric evaluation

To evaluate the suitability of the considered explanation designs and the overall satisfaction with the explainable decision support system, we conducted a second web-based survey. Like the previous survey, it was iteratively refined together with non-technical stakeholders in focus group sessions before it was sent out to all stakeholders to ensure that the survey was also understandable for non-technical users and that the explanations' design was as clear as possible, e.g., with descriptive labels and meaningful exemplary cases. It addressed the same stakeholders as the first survey. In total, 23 stakeholders participated (11 model consumers, six model builders, six model breakers). Again, we relied on a Likert-scale questionnaire. The first part of the survey focused on the considered representations of explanations (cf. Figures 10, 11), whereas the second part aimed at evaluating the decision support system as a whole.

#### 5.6.1 Preferences regarding the different explanation designs

**Figure 14:**
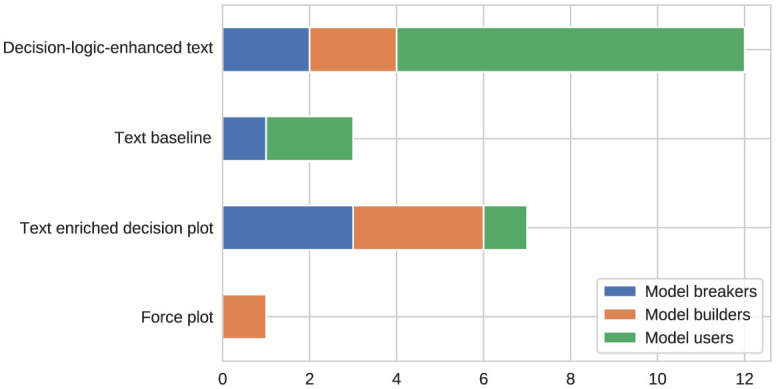
Number of stakeholders preferring the considered representation forms.

The survey asked all stakeholders to pick their favorite representation of explanations among the four considered variants. Figure 14 illustrates how many stakeholders preferred each of the available options. To identify any statistically significant deviations from a uniform distribution (*H*_0_:τ = 0.25), a right-sided binomial test was conducted for each option vs. the other options using a significance level of α = 0.05. In addition, the same test was applied to the overall preferences of all stakeholders. When focusing on model users, the *p*-values obtained for the decision-logic-enhanced text visualization were smaller than α, which leads to a rejection of the null hypothesis and indicates a statistically significant preference for this representation form. The same result was obtained when considering the overall preferences of all stakeholders. Furthermore, the Wald confidence intervals were (30.71%, 69.29%) for all stakeholders and (39.22%, 89.67%) when focusing on the model users. Because even the lower bound of these confidence intervals is greater than τ = 0.25, we consider the preference for the decision-logic-enhanced text design to be very strong. We also evaluated the comprehensibility and actionability of this preferred option using a Kruskal-Wallis test (with α = 0.05). According to the reuslts, all stakeholder groups agree that this particular form of explanations is understandable, easy to comprehend, and helps making decisions.

#### 5.6.2 Acceptance of the explainable decision support system

**Figure 15:**
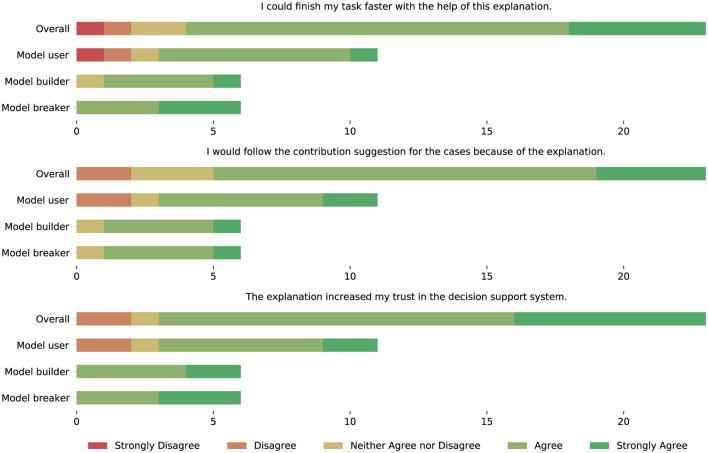
Frequency of agreement with statements regarding the trust in the eDSS and its impact on task performance per stakeholder group.

Besides the evaluation of different representation forms, we were also eager to testify if explanations based on feature importance are suited to increase the stakeholders' trust in the decision support system and if they believe that the system will have a positive impact on their task performance. Table 4 shows the questions included in our survey regarding these goals. The frequency distribution of the answers received for these questions are depicted in Figure 15. It should be noted that the null hypothesis of the non-parametric Kruskal-Wallis test, which states that the median is the same across all stakeholder groups, holds for all questions in Table 4, i.e., all stakeholders agree that the provided explanations increased their trust in the decision support system from which they believe that it will positively impact their task performance.

**Table 4:** Questions regarding the trust in the eDSS and its impact on task performance, as well as the median answers among all stakeholder groups.

**Statement**	**Answer**
The explanation increased my trust in the decision support system.	Agree
I would follow the contribution suggestion for the cases because of the explanation.	Agree
I could finish my task faster with the help of this explanation.	Agree

## 6 Discussion and conclusion

This paper presented a process model rooted in the XAI literature. It covers all the necessary steps for developing a *post-hoc* explanation system that enhances the transparency and trustworthiness of an existing black-box decision system. To demonstrate the usefulness of the proposed methodology, we applied it to a real-world problem in the automotive domain, which encompasses several characteristics like multiple stakeholder groups and a need for increased automation in conjunction with transparency, which are certainly present in other domains as well. Concretely, this study aimed to increase the trust and acceptance of stakeholders in an ML-based goodwill system. By following the process model, we were able to identify an XAI method, together with a suitable representation of the explanations it provides, that meets the requirements of different stakeholder groups. According to a final survey, all stakeholders agree that the selected and implemented XAI approach increases their trust in the decision system and can be expected to improve the performance of employees working with the system. From a design science research perspective, we believe that through our successful case study we have demonstrated our process model's *ease of use, efficiency, generality* and *operationality*, which are common evaluation criteria for method type artifacts (Sonnenberg and Vom Brocke, 2012). We further believe that our proposed process model can be transferred to other domains facing similar challenges, as presented in this study, such as multiple stakeholder groups and a tailored model requiring model-agnostic, *post-hoc* explanation methods for different stakeholder groups. In the following, we elaborate on some findings and limitations we identified during our study.

### 6.1 The importance of stakeholder involvement

The results of both surveys that we conducted in the course of our study emphasize the importance of stakeholder involvement in the XAI development process. Initially, we did neither anticipate the potential of XAI methods based on feature importance to meet their expectations nor their preference for text-based explanations.

Regarding the considered XAI methods, we expected that stakeholders favor rule-based explanations because a rule-based decision system is already used in the domain. Most probably, their choice for feature importance methods can be explained by the bad experiences with the decade-old and hence overly complex rule system, which might not be considered interpretable anymore. Moreover, although we expected counterfactual explanations to be less valuable for assessors working at the OEM, we saw them as an attractive solution for car dealers and their customers. After all, learning how changes in goodwill requests would affect the outcome of the goodwill process would allow them to maximize the compensation paid by the manufacturer. Finally, we expected that model breakers, i.e., managers, business specialists, and revisionists, would be more interested in a global perspective on the decision-making process than in analyzing individual goodwill requests. However, there appears to be a general preference across all stakeholders to inspect specific cases and draw conclusions from them instead of being provided with global explanations.

Another interesting outcome of our case study was the stakeholders' preference for text-based explanations over graphical representations, although the former are restricted to rankings of features and cannot convey information about their absolute importance. Nevertheless, many users, particularly model consumers, i.e., assessors responsible for goodwill decisions, preferred to be provided with textual information. These results may indicate that text-based feedback is perceived as natural by users without a technical background and can be understood more easily, even without previous training.

### 6.2 Effects on the acceptance of machine learning

The feedback we obtained from different interest groups via the previously discussed surveys indicates that their trust in the decision support system has increased. Compared to the initial reluctance of stakeholders to rely on a black-box model, the employment of XAI positively impacted the acceptance of ML-based technology. On the one hand, we attribute this newfound openness to the increase in transparency achieved through XAI. On the other hand, we believe that the involvement of stakeholders in the design and development process positively influenced their attitude toward the system.

Furthermore, we noticed that the possibility to analyze recommendations made by the ML model fosters discussions about the model's fairness and possible biases in human goodwill decisions. This suggests that XAI technologies can help to encourage fairness and increase awareness of unwanted biases in decision processes. However, increased trust in automated decision-making may also lead to over-reliance on the system, which is not desired in a high-stake business context built around the human-in-command principle. Instead, the goal should be an interplay between critically thinking human experts and the decision support system. As a countermeasure, the assessment process could be monitored to detect trends toward unilateral decisions that indicate algorithm aversion (Dietvorst et al., 2015) or automation bias (Lee and See, 2004).

### 6.3 Limitations and future work

Since the choice of suitable XAI approaches is very domain-specific, the process model proposed in this paper can only provide rough guidance. Consequently, it needs to be tailored to the specific use case, e.g., by considering appropriate explanation methods and presentation forms. Providing more guidance and even tool support to practitioners with regards to suitable explanation methods and designs depending on the domain, e.g., healthcare, finance, or the public sector, could be an interesting future avenue of research. As we have seen with the preference for textual explanation representation within this study, suitable methods and designs can be very domain-specific and contrary to common assumptions.

Moreover, the current process model only focuses on identifying, implementing, and evaluating *post-hoc* explanation methods that help to gain insights into an existing black-box model. In addition, future work may also deal with use cases where the goals of XAI should be considered from the start of the development process. In such cases, inherently interpretable white-box models can also play an important role and must therefore be taken into account.

The results of the first survey regarding the different explanation methodologies may also indicate that many stakeholders may not have fully understood the differences between the various explanation methods. This is evidenced by the agreement that all explanations are useful, but little difference in preferences among the methods. The purely textual web-based survey format could have been a limiting factor in this case. The second survey, which incorporated both textual and visual representations of the explanation methods, led to more nuanced results. This suggests that presenting explanations in a more tangible way, with more concrete domain-specific examples that stakeholders can relate to, appears beneficial.

In general, gathering feedback from human stakeholders remains a cumbersome and challenging task due to their limited availability and ML/XAI expertise, which may also explain the primary usage of XAI by developers (Bhatt et al., 2020). Hence, there is a severe risk of biased feedback results originating from poorly designed XAI surveys, leading to misguided XAI systems. Pre-validating designs and surveys in focus groups, as done in our study, may be a way to prevent larger misconceptions and misunderstandings among stakeholders. However, automating, validating, and easing the collection of user feedback may be an important avenue for future research (Confalonieri and Alonso-Moral, 2024), as collecting stakeholder feedback is of utmost importance when developing XAI systems. Guidance in terms of XAI survey creation, visualization, and validation could reduce the risk of misconceptions and misguided XAI systems.

In terms of stakeholder segmentation, as discussed in Section 5.1, a more structured and fine-grained approach may also be beneficial, particularly to further split the model breaker stakeholders into more distinct interest groups. Model breakers usually encompass several interest groups, each of which may have distinct explanation needs, whereas the builder and user groups appear more homogeneous. Due to time and resource constraints, user segmentation was not carried out to the full extent in this study.

In terms of computational efficiency, the utilization of Kernel SHAP was not an issue in this study, where explanations could be generated in an asynchronous way. However, for applications that require real-time explanations, the usage of Kernel SHAP could be problematic due to the high memory usage and runtime as demonstrated in Section 5.5.3. Here, more efficient SHAP estimators may be required.

## Data Availability

The data analyzed in this study is subject to the following licenses/restrictions: private company owned data. Requests to access these datasets should be directed to stefan.sh.haas@bmwgroup.com.

## Footnotes

^1^ https://github.com/slundberg/shap
